# Genome Sequences of Three Common Bacterial Isolates from Wastewater from the Water Processor Assembly at the International Space Station

**DOI:** 10.1128/mra.01189-22

**Published:** 2023-01-04

**Authors:** Yo-Ann Velez Justiniano, Chae Hee Lim, Darren S. Dunlap, Tatyana A. Sysoeva

**Affiliations:** a Aerodyne Industries, LLC, Cape Canaveral, Florida, USA; b Department of Biological Sciences, The University of Alabama in Huntsville, Huntsville, Alabama, USA; c Huntsville Central Laboratory, Boeing Research & Technologies, The Boeing Company, Huntsville, Alabama, USA; University of Delaware

## Abstract

Bacteria from the genera *Burkholderia*, *Ralstonia*, and *Methylobacterium* were consistently detected in water of the life support systems at the International Space Station. Here, we report complete genomes of recent isolates that are representative of these genera to support future studies in biofilm and wastewater treatment in space habitats.

## ANNOUNCEMENT

The International Space Station (ISS) relies on the water recovery system (WRS), which provides around 90% of all ISS water by recycling urine and condensate from the air humidity (1). The WRS encompasses urine recycling hardware, a containment wastewater tank, a water-processing assembly, and a potable water dispenser (1). A few incidents showed that the WRS is susceptible to microbial biofilm growth, which can compromise its function (2). Bacterial species from the Burkholderia cepacia complex and *Ralstonia* and *Methylobacterium* genera were commonly cultured from the WRS wastewater and cabin condensate (3). Similarly, on Earth, representatives of the same genera are found contaminating water sources, water-based pharmaceuticals, water dispensers, and potable water, forming biofilms that compromise water quality and the functioning of the water systems (4–6). To start detailed characterization of wastewater ISS isolates and to compare those with their counterparts found at normal gravity, we used isolates obtained from ISS samples by the Boeing Huntsville laboratory.

Water samples from the process water line immediately downstream of the wastewater tank in the wastewater orbital replacement unit (ORU) were collected in Teflon bags via adapter hosing on 11 and 19 November 2015 and were shipped on 30 November 2015. Within 24 h at 4°C after receipt of the samples in Huntsville, Alabama (16 December 2015), the samples were filtered through 0.45-μm cellulose filters; they were then incubated on Reasoner’s 2A (R2A) agar at 28°C for 7 days. The colonies obtained were streaked two times to confirm purity and then were used for preparation of −80°C freezer glycerol stocks and further analysis. Initially, isolates were identified by fatty acid methyl ester (FAME) analysis with the Sherlock microbial identification system (part number 2080; MIDI, Newark, DE, USA) using the MIDI FAME standard and Stenotrophomonas maltophilia as quality controls. For DNA extraction, samples were streaked on R2A agar at 25°C for 24 h, and then a single colony was grown overnight in liquid LB at 25°C. DNA extraction kits, i.e., ZymoBIOMICS 96 MagBead DNA and DNeasy blood and tissue kits, were used for short-read Illumina sequencing and long-read Nanopore sequencing, respectively. DNA concentrations were measured with a Qubit fluorometer with Invitrogen Qubit double-stranded DNA broad-range assay kit and a NanoDrop spectrophotometer. Short-read sequencing libraries were prepared by following procedures in the Nextera DNA Flex library preparation kit to obtain paired-end 150-bp reads using the Illumina NovaSeq sequencer. The Oxford Nanopore Technologies MinION Mk1C instrument was used with the SQK-LSK110 kit protocol, a R9.4.1 flow cell, 72 h of data collection, and default automatic base calling with Guppy v3.2.4. Illumina adapters and reads smaller than 70 bp were removed with Trimmomatic v0.33 (7), and long-read adapters were removed with Porechop (8). All sequencing data were checked with FastQC v0.11.9 (9). Genomes of the isolates were established *de novo* using hybrid assembly via the Unicycler pipeline v0.4.8 (10) run in normal mode for Ralstonia insidiosa and Methylobacterium organophilum and in bold mode for Burkholderia contaminans data. All other software was used with default settings.

Genome contamination and species identities were assessed using ContEst16S (11), and no contamination was found. The 16S RNA gene sequences were then used in NCBI BLASTn searches to determine similarity. The most similar species in the BLASTn searches for all three were Burkholderia contaminans, Ralstonia insidiosa, and Methylobacterium organophilum. Genomes were annotated by the automated Prokaryotic Genome Annotation Pipeline (PGAP) v5.1 upon NCBI submission (12). Final assemblies contained all complete circular chromosomes and plasmids summarized in Table 1. These genomes will facilitate analyses of biofilm contamination of the WRS system at the ISS, as well as finding ways to counteract this contamination.

**TABLE 1:** Genome sequencing of ISS wastewater bacterial isolates

Isolate	GenBank accession no.	SRA accession no. (output [no. of reads])		Median genus GC content (%)	Isolate GC content (%)	Total genome size (bp)	No. of chromosomes (size [bp])	No. of plasmids (size [bp])
		Illumina sequencing	Nanopore sequencing					
Burkholderia contaminans B17-01563-1	GCA_022533485.1	SRX14394785 (20.23 Gbp [13.7 million])	SRX14394786 (144 Mbp [144.7 thousand])	66.7	66.30	8,617,690	3 (3,627,156; 3,278,817; 1,529,712)	1 (182,005)
Ralstonia insidiosa B19 15-1563-3	GCA_022631215.1	SRX14394783 (4.1 Gbp [13.6 million])	SRX14394784 (452.6 Mbp [92.9 thousand])	63.7	63.29	6,325,797	2 (3,722,972; 1,903,019)	3 (310,422; 298,156; 91,228)
Methylobacterium organophilum B15 WPA-B	GCA_022533465.1	SRX14394781 (4.1 Gbp [13.7 million])	SRX14394782 (185.4 Mbp [22.2 thousand])	68.95	69.80	5,319,780	1 (5,247,103)	2 (37,085; 35,592)

### Data availability.

The genome sequences discussed here and their raw data were deposited under the BioProject accession number PRJNA805242. The GenBank and Sequence Read Archive (SRA) accession numbers are given in Table 1.
